# Improving the thermal resistance of fluorescent CsPb(Br,I)_3_ perovskite quantum dots by surface modification with perfluorodecanoic acid

**DOI:** 10.1098/rsos.220475

**Published:** 2022-08-24

**Authors:** Yoshiki Iso, Momoko Eri, Risako Hiroyoshi, Kensho Kano, Tetsuhiko Isobe

**Affiliations:** Department of Applied Chemistry, Faculty of Science and Technology, Keio University, 3-14-1 Hiyoshi, Kohoku-ku, Yokohama 223-8522, Japan

**Keywords:** caesium lead halide perovskite quantum dot, phosphor, photoluminescence, thermal resistance, heat degradation, surface ligand modification

## Abstract

CsPb(Br,I)_3_ quantum dots (QDs) show application potential for optoelectronic devices. However, their thermal degradation is a significant problem. In this work, the effects of perfluorodecanoic acid (PFDA) modification on the photoluminescence (PL) and thermal resistance of CsPb(Br,I)_3_ QDs were evaluated. The PL intensity of oleic-acid-modified quantum dots (OA-QDs) in toluene decreased drastically upon heating at 100°C. The PL quantum yield of the QDs increased from 69.6% to 77.4% upon modification with PFDA. Furthermore, the PL intensity of the QDs modified with PFDA (PFDA-QDs) increased to 140.6% upon heating, because of the reduction of surface defects upon adsorption of PFDA and its optimized adsorption state. A solid-film PFDA-QDs sample heated at 80°C for 4 h showed temporary PL enhancements for the OA-QDs and PFDA-QDs films to 445% and 557% of their initial values, respectively, upon heating for 0.25 h. This was attributed to the optimized adsorption states of the surface ligands. PFDA-QDs film maintained 354% after 4 h of heating, whereas that of OA-QDs film was 104%. Thus, PFDA modification enhances PL intensity and suppresses PL degradation under heating, which is important for wavelength converters for optoelectronic device applications.

## 1. Introduction

CsPbX_3_ (X = Cl, Br, I) perovskite quantum dots (QDs) have attracted extensive research attention owing to their excellent properties, including their bandgap (*E*_g_) tunability (by halide composition), narrow luminescence peaks and high photoluminescence (PL) quantum yields (PLQYs) [1,2]. Accordingly, CsPbX_3_ QDs have been applied in LEDs [3–10], wide-gamut displays [11–14], lasers [15–17], scintillators [18] and photovoltaic devices [19–24]. However, like organic–inorganic perovskite QDs, all-inorganic CsPbX_3_ QDs are insufficiently durable for practical use yet. Thus, the development of strategies for improving their resistance to degradation by heat, excitation light, ambient air and/or moisture are required. Accordingly, numerous reports on improving the stability of CsPbX_3_ QDs by various techniques [25], including B-site doping (for ABX_3_ perovskites) [26,27], surface-defect elimination [28,29], the use of core–shell architectures [30] and matrix encapsulation [31], have been reported.

The current authors have focused on the effects of surface ligands on fluorescent QDs. Surface ligands protect QDs from surrounding molecules, passivate surface defects that cause non-radiative relaxation and provide dispersibility in solvents. Therefore, the state of adsorbed surface ligands is an important factor for the quality of fluorescent QDs. Oleic acid (OA) is generally used as a surface ligand in the traditional preparation of CsPbX_3_ QDs. However, its desorption from the QDs causes significant degradation of their PL properties [32,33]. The acid dissociation constant (p*K*_a_) of an alkylcarboxylic acid indicates its frequency of deprotonation and thus its adsorptivity as a surface ligand, because carboxylic acids coordinate to crystal surfaces in the deprotonated state. Fluorocarboxylic acids, which have electron-withdrawing fluorine atoms, exhibit lower p*K*_a_ values than OA [34]. The p*K*_a_ values of perfluorodecanoic acid (PFDA) and OA are 2.58 and 6.2, respectively [35,36]. Thus, the deprotonated state of PFDA is more stable than that of OA, so it should more readily adsorb onto a QD surface.

Monohalide CsPbX_3_ QDs are not suitable for certain optoelectronic device applications. Instead, desired PL properties are realized by precisely tuning the composition of mixed-halide QDs. I-doped CsPbBr_3_ QDs (typically represented by the label CsPb(Br,I)_3_ QDs) realize green emission, the colour of which is very close to the vertex of chromaticity coordinate in the Rec.2020 standard for wide-colour-gamut displays [13,37]. However, mixed-halide QDs can be subject to partial decomposition and/or dissolution, changing the halide compositional ratio and thus leading to shifts in their absorption and emission wavelengths.

In this work, PFDA modification of OA-adsorbed CsPb(Br,I)_3_ QDs was performed to investigate its effect on thermal resistance. Toluene dispersions of the as-prepared QDs and PFDA-modified QDs were heated at 100°C. Decanoic acid (DA) modification was also performed as a control procedure. Furthermore, solid films of the QDs obtained by vacuum drying were also evaluated.

## 2. Experimental

### 2.1. Materials

Cs_2_CO_3_ (99.99%) was purchased from Mitsuwa Pure Chemical. PbO (99.9%), toluene (>99.5%), acetone (>99.5%) and methyl acetate (>99.5%) were purchased from Kanto Chemical. OA (>85.0%), tetra-*n*-octylammonium bromide (>98.0%), tetra-*n*-octylammonium iodide (>98.0%) and DA (>98.0%) were purchased from Tokyo Chemical Industry. PFDA (98%) was purchased from Sigma-Aldrich. Toluene, acetone and OA were dehydrated over molecular sieves (3Å 1/8, FUJIFILM Wako Pure Chemical) prior to use.

### 2.2. Synthesis of CsPb(Br,I)_3_ QDs and surface modification

The synthesis of CsPb(Br,I)_3_ QDs by the ligand-assisted reprecipitation (LARP) method expanded upon those detailed previously [34]. Cs_2_CO_3_ (163 mg), PbO (223 mg) and 5 ml of OA were mixed and heated at 160°C to obtain a clear solution, which was then dehydrated for 30 min at 120°C. After cooling and adding 5 ml of toluene, 1 ml of this solution was added to a glass vessel containing 15 ml of toluene under vigorous stirring at room temperature. A clear halide solution was prepared by heating a mixture of tetra-*n*-octylammonium bromide (38 mg), tetra-*n*-octylammonium iodide (18 mg), 5 ml of OA and 2 ml of toluene at approximately 50°C. Here, the nominal ratio of Br : I was 70 : 30. The halide solution was swiftly added to the glass vessel at room temperature to synthesize CsPb(Br,I)_3_ QDs. After 10 s, the QDs were precipitated by adding 50 ml of acetone and then collected by centrifugation at approximately 19 000×*g* (13 000 r.p.m. using a rotor of 10 cm in radius) for 5 min. The collected QDs were redispersed in toluene under ultrasonication and stirring to prepare an OA-QDs dispersion. The concentration was adjusted with reference to absorbance as described in a later section.

DA-QDs and PFDA-QDs dispersions were prepared by adding DA and PFDA, respectively, to the OA-QDs dispersion to achieve a concentration of 0.06 mmol l^−1^. The prepared QD dispersions were sealed and stored under ambient conditions in the dark. To evaluate their thermal stabilities, the QD dispersions were heated at 100°C for 4 h in an incubator (HB-100, Taitec) with shaking at 60 r.p.m., and then cooled to room temperature before analysis.

### 2.3. Preparation of solid-film samples

**Figure 1.**
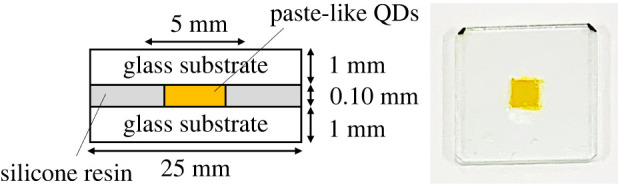
Schematic and photograph of a QD-film sample.

Film samples were prepared to evaluate the properties of solid QDs. 15 ml of toluene was mixed with 1 ml of metal-ion solution and halide solution to nucleate CsPb(Br,I)_3_ QDs. After 10 s, 200 µl of a toluene solution of PFDA at 10.8 mg l^−1^ was added to the obtained QD dispersion and aged for 1 min. PFDA-modified QDs were precipitated by adding 50 ml of methyl acetate and collected by centrifugation at approximately 19 000×*g* (13 000 r.p.m. using a rotor of 10 cm in radius) for 5 min. The precipitation was dried under vacuum for 12 h to obtain a paste-like solid sample of the PFDA-QDs. Paste-like OA-QDs were also prepared in the same way without adding PFDA. QD-film samples were prepared from the paste-like materials using glass substrates and silicone spacers, as shown in figure 1. To evaluate thermal stability, the film samples were heated at 80°C and 45% relative humidity for 4 h in a thermo-hygrostat (IG401, Yamato Scientific). It should be noted that the temperature used was the upper limit of the apparatus.

### 2.4. Characterization

X-ray diffraction (XRD) profiles were measured on an X-ray diffractometer (Rint-2200, Rigaku) using monochromatic CuK*α* radiation. Elemental compositions were determined using a wavelength dispersive X-ray fluorescence (XRF) analyser (ZSXmini II, Rigaku). Herein, wavelength dispersive mode was chosen to obtain reliable elemental compositions. For the XRD and wavelength dispersive XRF measurements, the centrifuged QDs were vacuum dried overnight. Fourier transform infrared (FT-IR) absorption spectra of QDs in pressed KBr discs were measured using a spectrometer (FT/IR-4200, JASCO). The particle images were captured with a field-emission transmission electron microscope (TEM; Tecnai G^2^, FEI). Samples for TEM were prepared by drying a drop of the QD dispersion on a carbon-reinforced collodion-coated copper grid (COL-C10, Oken Shoji) overnight. Energy dispersive XRF analysis attached to the TEM was performed. Particle size distributions were determined by measuring 100 particles in TEM images at random. Evaluation of optical properties of the heated samples was performed after cooling to room temperature. Thermal analysis of the film samples was performed on a thermogravimetry analysis (TGA) instrument (Thermo Plus TG8120, Rigaku) in an Ar flow of 500 ml min^−1^ at a heating rate of 10°C min^−1^. The UV–visible absorption spectra of the dispersions were measured using a UV/visible absorption spectrometer (V-750, JASCO). To ensure analysis at the same concentration, the net absorbance of the dispersion before heating was adjusted to 0.35 at 400 nm. The shown absorbance data are the net values calculated by subtracting the blank data for the pure solvent from the sample data. Tauc plots were prepared to determine the *E*_g_ values of the QDs according to equation (2.1) [38]:

2.1 $${\left(\alpha h\nu \right)}^{1/n}=A(h\nu -E_{g}),$$

where *α* is the absorbance, *h* is the Planck constant, *ν* is the frequency and *A* is a constant. The value of *n* was 0.5 because cubic CsPbX_3_ are direct-transition-type semiconductors [39]. The PL spectra were recorded on a fluorescence spectrometer (FP-6500, JASCO). Herein, an ethylene glycol solution of rhodamine B (5.5 g l^−1^) and a standard light source (ESC-333, JASCO) were used for calibration of each spectral response. Absolute PLQY values were determined using a quantum efficiency measurement system (QE-2000-311C, Otsuka Electronics).

## 3. Results and discussion

### 3.1. Effects of surface modification by PFDA on the thermal stability of CsPb(Br,I)_3_ QD dispersions

Judging from the XRD profile (electronic supplementary material, figure S1), the as-prepared CsPb(Br,I)_3_ QDs have a cubic crystal structure. The peaks in the XRD profile are between those corresponding to cubic CsPbBr_3_ and cubic CsPbI_3_. The Br : I ratio from wavelength dispersive XRF is 32.2 : 68.8, which is close to the nominal ratio of 30 : 70. Judging from the FT-IR spectra for the solid-film samples, PFDA-QDs contain PFDA with remaining OA (see electronic supplementary material, figure S2).

**Figure 2.**
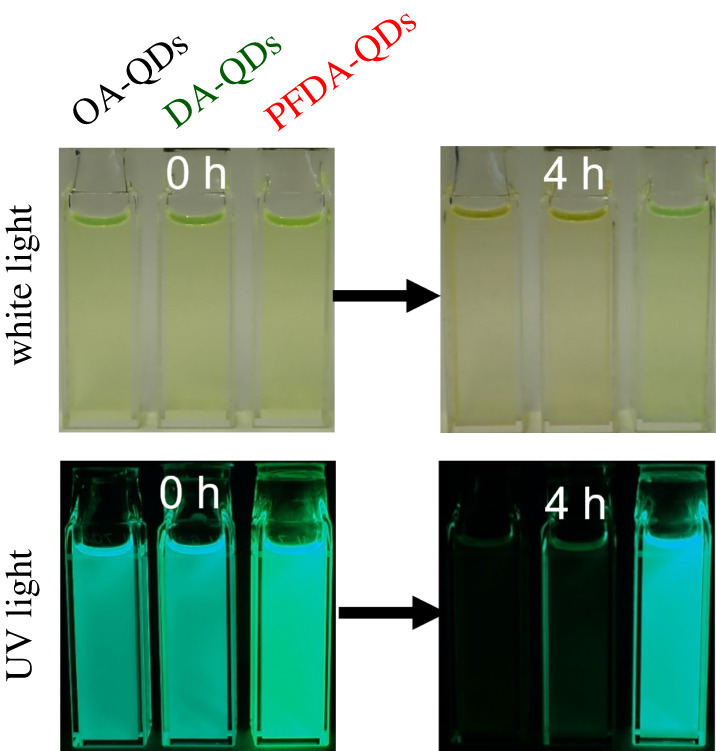
Photographs of QD dispersions under white light and 365 nm UV light before and after heating at 100°C for 4 h.

Figure 2 shows photographs of the QD dispersions before and after heating at 100°C for 4 h. The as-prepared PFDA-QDs dispersion shows brighter luminescence compared with the other dispersions. The PL peak for the PFDA-QDs dispersion is observed at 515.0 nm, which is a longer wavelength than those of the OA-QDs and DA-QDs dispersions (504.2 and 504.1 nm; electronic supplementary material, figure S3). Although the mechanism of the PL redshift is unclear, the adsorbed PFDA ligands might have a greater influence on the band structure of the QDs than the OA and DA ligands. Furthermore, PFDA modification enhances the PLQY from that of the OA-QDs dispersion (69.6%) to 77.4%, while DA addition results in no improvement. These results indicate that the strongly and frequently adsorbed PFDA ligands decrease the amount of surface defects on the CsPb(Br,I)_3_ QDs.

After heating at 100°C for 4 h, a yellow precipitation of aggregated QDs is observed under white light for the OA-QDs and DA-QDs, while the PFDA-QDs show no change visible to the naked eye. Furthermore, extinction of the green emission under UV light occurs for the OA-QDs and DA-QDs, while the PFDA-QDs maintain their strong luminescence, even upon heating. These results indicate that surface modification with PFDA stabilizes the dispersibility and PL of the QDs.

**Figure 3.**
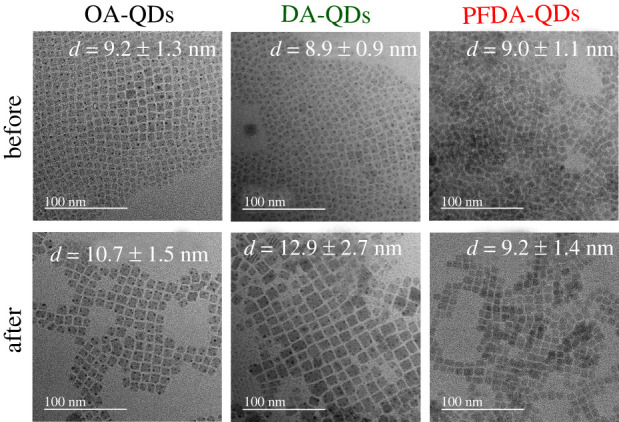
Particle morphologies observed by TEM for OA-QDs, DA-QDs and PFDA-QDs before and after heating at 100°C for 4 h.

Figure 3 shows TEM images of the QDs before and after heating at 100°C for 4 h (see also high-resolution TEM images in electronic supplementary material, figure S4). Particle size distributions were measured to determine the mean sizes of QDs (see electronic supplementary material, figure S5). The mean sizes of the OA-QDs, DA-QDs and PFDA-QDs are 9.2 ± 1.3, 8.9 ± 0.9 and 9.0 ± 1.1 nm; therefore, significant change was not observed by DA and PFDA additions. The mean size of the OA-QDs increases to 10.7 ± 1.5 nm during heating because of the dissolution and precipitation process. Crystal growth was also observed for the DA-QDs. However, PFDA modification maintains the size at 9.2 ± 1.4 nm, even after heating. Thus, the rigid surface adsorption of PFDA suppresses the dissolution and precipitation process. Furthermore, this also accounts for the excellent dispersibility of QDs upon heating (figure 2), because steric hindrance by the strongly adsorbed PFDA ligands prevents QD aggregation.

**Figure 4.**
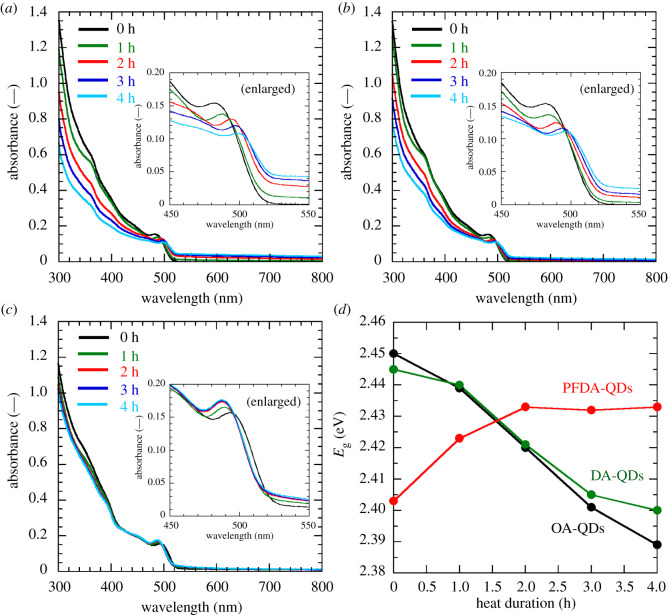
Changes in (*a–c*) UV–visible absorption spectra and (*d*) estimated *E*_g_ for the QD dispersions upon heating at 100°C. (*a*) OA-QDs, (*b*) DA-QDs and (*c*) PFDA-QDs.

Figure 4*a–c* shows time-evolution UV–visible absorption spectra upon heating. The change in the spectra for the PFDA-QDs dispersions is smaller than those for the OA-QDs and DA-QDs dispersions. Heating the OA-QDs and DA-QDs dispersions for 4 h causes a slight redshift of the absorption edge and an increase in the baseline absorbance in the 500–800 nm range. This may be explained by increased light scattering due to the growth and aggregation of QDs and corresponds to the appearance of yellow aggregates in the OA-QDs and DA-QDs dispersions under white light.

*E*_g_ values were determined from Tauc plots of the UV–visible absorption data (see electronic supplementary material, figures S6–S8). Figure 4*d* shows the changes in the estimated *E*_g_ values. OA-QDs and DA-QDs dispersions show decreases in *E*_g_ upon heating. This is due to the quantum size effect that accompanies QD growth. By contrast, the PFDA-QDs dispersion shows an increase in *E*_g_ upon heating. This cannot be explained by the quantum size effect because no change in crystal size is observed in the TEM images. The *E*_g_ value of CsPb(Br,I)_3_ QDs also varies with halide composition. The increase in *E*_g_ may result from the decrease in I/Br ratio, which occurs because of the preferential dissolution of iodine ions. Regrettably, precise elemental analysis by wavelength dispersive XRF was impossible because the PFDA-QDs could not be collected from the dispersion even after heating. Moreover, a reliable elemental composition was not determined by the energy dispersive XRF analysis attached to the TEM because of peak overlaps (see electronic supplementary material, figure S9).

**Figure 5.**
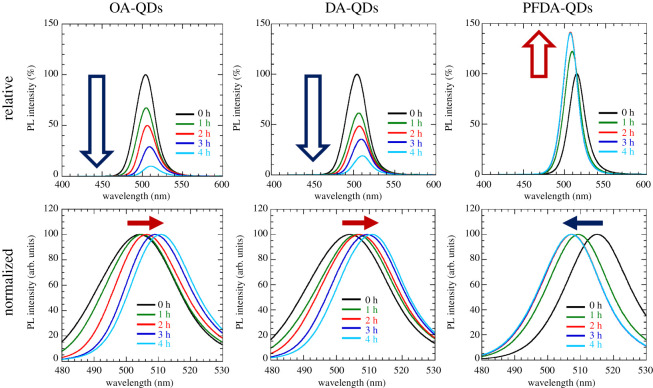
Changes in relative and normalized PL spectra for toluene dispersions of OA-QDs, DA-QDs and PFDA-QDs heated at 100°C. *λ*_ex_ = 400.0 nm.

**Figure 6.**
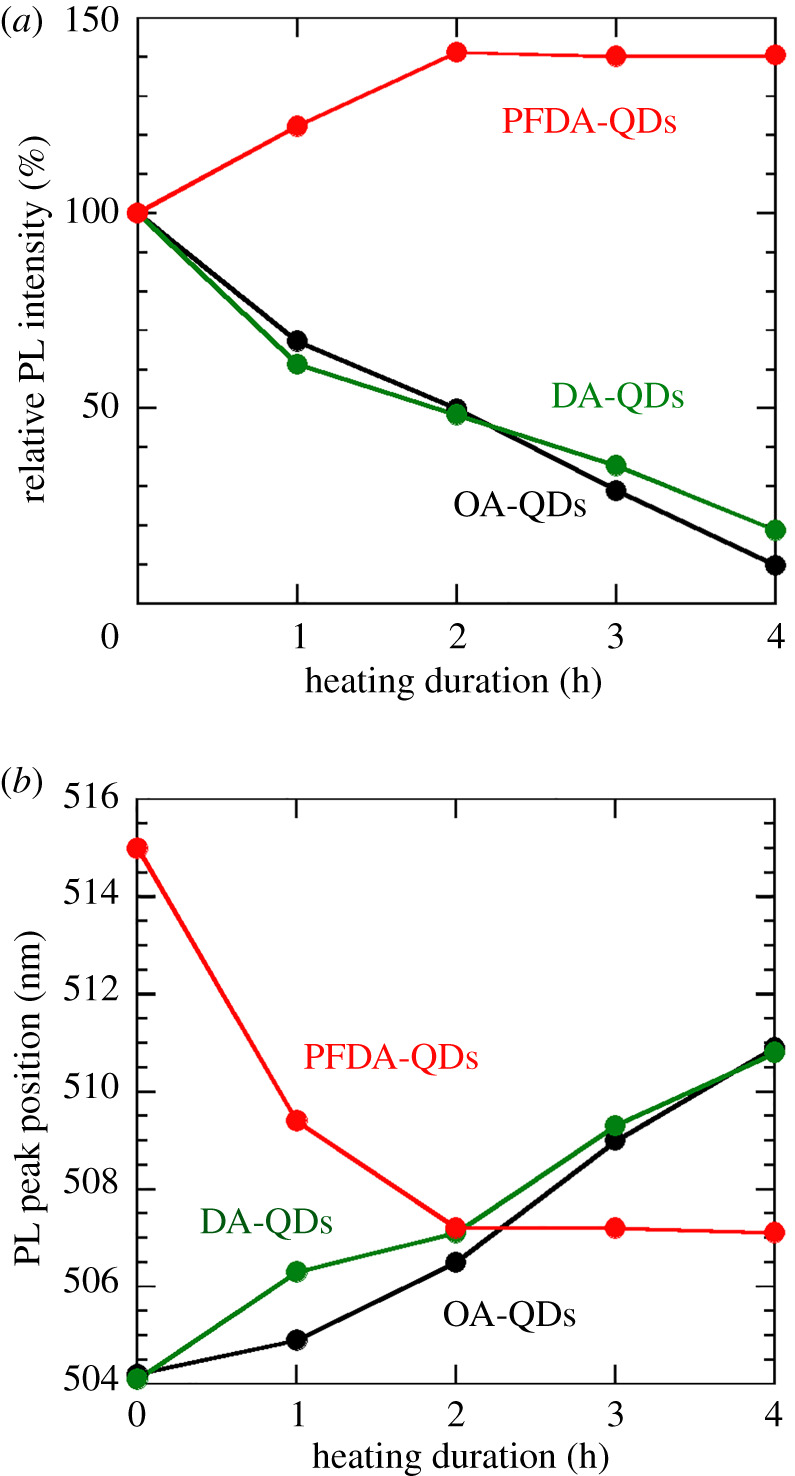
Changes in (*a*) PL peak intensity and (*b*) position (from data shown in figure 5).

Figure 5 shows the PL spectra of the dispersions measured at a room temperature. Here, the initial PL intensities were normalized to 100%. Changes in PL intensity and peak position with time are shown in figure 6. OA-QDs and DA-QDs dispersions show monotonic decreases in PL intensity to 9.8% and 18.8% upon heating, whereas the PFDA-QDs dispersion presents the highest PL intensity and no quenching throughout the experiment. Interestingly, the PL intensity increases until 2 h of heating, reaching 140.6%, indicating that a reduction in the number of surface defects, which cause non-radiative relaxation, occurs because of rapid adsorption of PFDA and its optimized adsorption state. Furthermore, the heat-ageing of QDs synthesized at room temperature by the LARP method might contribute to the improved PL intensity. The PL peak position for the OA-QDs dispersion shifts from 504.2 to 510.9 nm, while that for the DA-QDs dispersion also shows a redshift (from 504.1 to 510.8 nm). Conversely, a blueshift from 515.0 to 507.1 nm is observed for the PFDA-QDs dispersion. These shifts correspond to the changes in the absorption edges observed in figure 4. Therefore, the PL peak shifts can be explained by the same mechanisms used to rationalize the changes in *E*_g_.

### 3.2. Effects of surface modification by PFDA on the thermal stability of CsPb(Br,I)_3_ QD films

**Figure 7.**
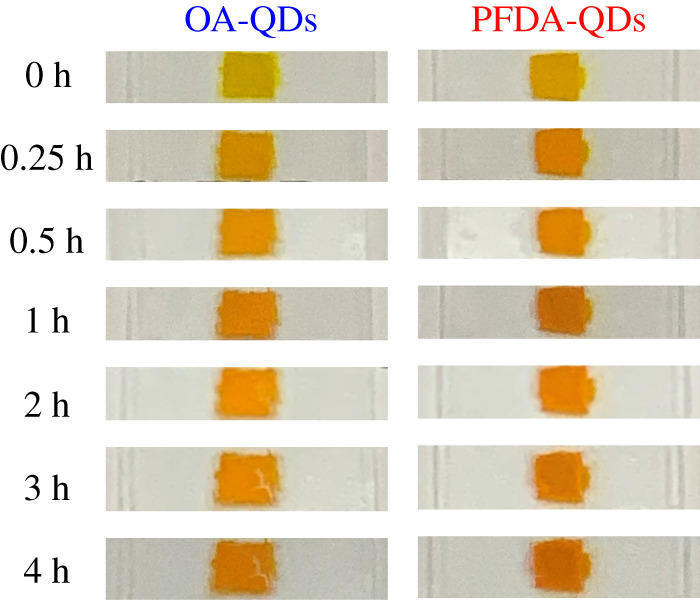
Photographs of QD films before and after heating at 80°C.

Figure 7 shows photographs of OA-QDs and PFDA-QDs films captured under white light during heating at 80°C for 4 h. The sample colour for both films under white light changes from yellow to orange. The photographs captured under 365 nm UV light show no specific change trend in PL intensity under UV irradiation because of the automatic image adjustment of the digital camera (see electronic supplementary material, figure S10). Accordingly, reliable PL intensities were measured using a fluorescence spectrometer, as shown later. It should be noted that no significant PL quenching was observed by the naked eye.

**Figure 8.**
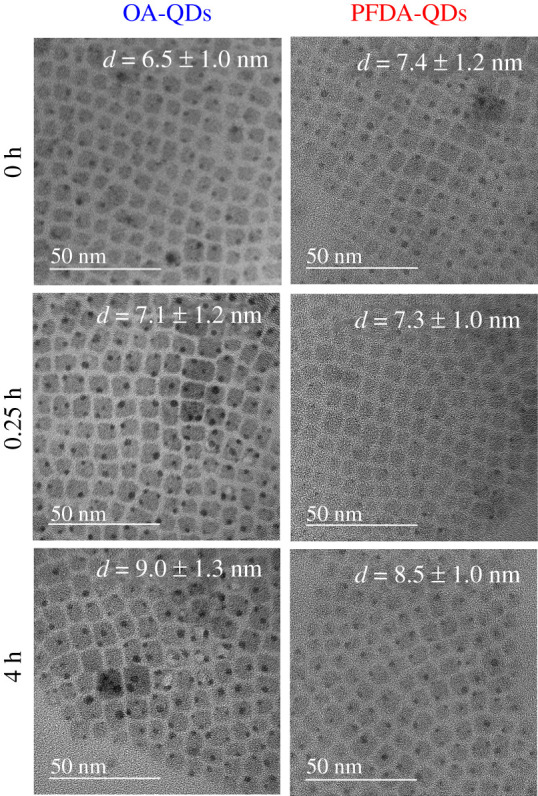
Particle morphologies of OA-QDs and PFDA-QDs films observed by TEM before and after heating at 80°C.

TEM images of the film samples are shown in figure 8. Average particle size values were estimated from the size distributions (see electronic supplementary material, figure S11). The OA-QDs film shows a 38% increase in average size (from 6.5 ± 1.0 to 9.0 ± 1.3 nm) upon heating for 4 h. The film samples comprise paste-like QDs where many organic molecules surround the inorganic QDs. Thus, the crystal growth observed here may be caused by dissolution and reprecipitation through ion transfer among the organic molecules. Conversely, the PFDA-QDs film shows a 15% increase (from 7.4 ± 1.2 to 8.5 ± 1.0 nm) upon heating for 4 h. The rigid surface modification of PFDA prevents dissolution of the QDs, resulting in suppressed crystal growth during heating. TGA also exhibited improved thermal stability of PFDA-QDs (see electronic supplementary material, figure S12).

**Figure 9.**
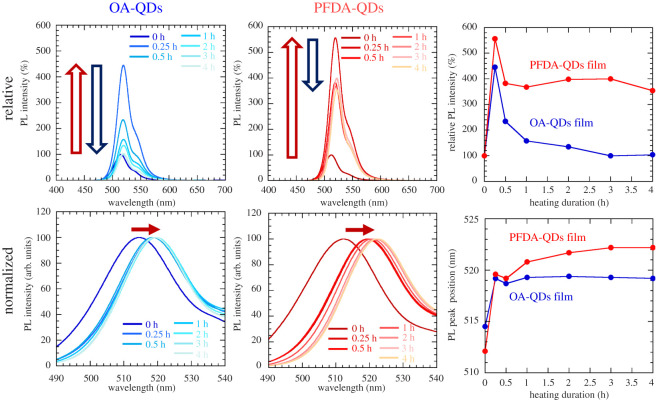
Changes in the relative and normalized PL spectra of QD films during heating at 80°C. The corresponding changes in PL peak intensity and position are also plotted.

**Figure 10.**
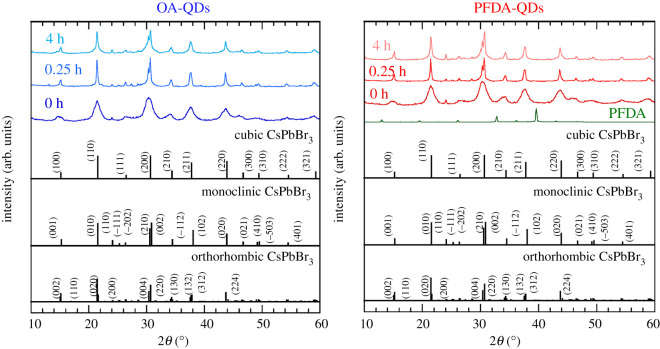
Changes in the XRD profiles of QD films during heating at 80°C. ICDD card data for cubic, monoclinic and orthorhombic CsPbBr_3_ (nos. 00-054-0752, 00-054-0751 and 01-072-7929) are also shown.

Figure 9 shows the PL spectra of the films measured at a room temperature. The time evolution of peak intensity and the peak shift are also summarized. The initial PL intensities before heating are normalized to 100%. A shoulder peak at longer wavelength is observed for the dried film samples, as reported in our previous works [32,33,40]. This may be explained by the photon recycling effect [41]. The PL intensities for the OA-QDs and PFDA-QDs increase to 445% and 557%, respectively, upon heating for 0.25 h, but they both decrease upon further heating. This PL increase may be attributed to the optimized adsorption states of ligands and improved crystallinity upon heat-ageing. Judging from the XRD profiles in figure 10, the cubic phase of CsPb(Br,I)_3_ is converted to monoclinic or orthorhombic phases. Regrettably, the monoclinic and orthorhombic phases could not be accurately distinguished because their patterns are very similar. However, they are emissive and non-emissive phases, respectively. The decrease in PL intensity may be caused by a partial phase transition to the non-emissive phase. The final PL intensities of the OA-QDs and PFDA-QDs films at 4 h are 104% and 354%. Therefore, PFDA modification improves the durability of the QDs.

The PL peak position of the OA-QDs film is shifted from 514.5 to 519.2 nm, similar to the PL redshift of the OA-QDs dispersion. However, the peak position of the PFDA-QDs film shows a redshift from 512.1 to 522.2 nm, while that of its dispersion shows a blueshift. The absence of solvent prevents the preferential dissolution of iodine ions, which results in no change in the Br/I ratio of the QDs in the film. Therefore, the PL redshift is probably observed because of quantum size effects associated with the crystal growth, as observed in the TEM analysis. It should be noted that the influence of the phase transition on the PL peak shift should be negligible because the *E*_g_ values for the cubic and monoclinic phases are similar [42].

## 4. Conclusion

In this work, the influence of PFDA modification on CsPb(Br,I)_3_ QDs prepared by the LARP method was investigated. Significant aggregation and quenching of green emission under UV light were observed for toluene dispersions of OA-QDs and DA-QDs upon heating at 100°C for 4 h, while the PFDA-QDs dispersion maintained its strong luminescence without precipitation, revealing that PFDA stabilizes the dispersibility and PL of these QDs through surface modification. The rigid adsorption of PFDA also contributed to the suppression of crystal growth, as observed by TEM analysis. Heat treatment was observed to decrease the *E*_g_ of OA-QDs and DA-QDs in toluene because of crystal growth upon dissolution and reprecipitation. Conversely, *E*_g_ for the PFDA-QDs increased during heating, indicating a decrease of I/Br ratio in the QDs by the preferential dissolution of iodine ions. Improved thermal resistance upon PFDA modification was also observed for the dried QD film. Furthermore, the final PL intensity of the PFDA-QDs film after heating at 80°C for 4 h was 354% of the initial value. These improvements to PL and thermal resistance by PFDA modification have great significance in terms of the practical application of CsPb(Br,I)_3_ perovskite QDs to optoelectronic devices that heat up during use, such as LEDs and wide-gamut displays.

## Data Availability

The data are provided in the electronic supplementary material [43].
